# AI for One Welfare: the role of animal welfare scientists in developing valid and ethical AI-based welfare assessment tools

**DOI:** 10.3389/fvets.2025.1645901

**Published:** 2025-08-05

**Authors:** Borbala Foris, Kehan Sheng, Christian Dürnberger, Maciej Oczak, Jean-Loup Rault

**Affiliations:** ^1^Centre for Animal Nutrition and Welfare, Clinical Department of Farm Animals and Food System Science, University of Veterinary Medicine Vienna, Vienna, Austria; ^2^Animal Welfare Program, Faculty of Land and Food Systems, The University of British Columbia, Vancouver, BC, Canada; ^3^Messerli Research Institute, University of Veterinary Medicine Vienna, Vienna, Austria; ^4^Precision Livestock Farming Hub, Clinical Department of Farm Animals and Food System Science, University of Veterinary Medicine Vienna, Vienna, Austria

**Keywords:** generative AI, large multimodal model, AI alignment, Welfare Quality, animal interests, precision livestock farming

## Abstract

The increasing use of artificial intelligence (AI) in livestock farming is accelerating the development of automated welfare assessment tools, particularly with advancement in generative AI such as large multimodal models (LMMs). Yet, animal welfare scientists have rarely been involved in the development process of these tools or their subsequent adaptation within the field. Here, we discuss possible roles for animal welfare scientists in the development and validation of AI-based welfare assessment tools. We first examine key uncertainties that emerge during development, including the selection of relevant, valid and reliable welfare indicators and gold standards, hardware and software solutions for data collection, methods for integrating multiple welfare indicators, and the real-world impact of automated welfare assessment tools. Second, we demonstrate the use of LMMs to assess welfare based on a case study using dairy cow cleanliness. Finally, we consider the practical implementation of AI-based welfare assessment and discuss potential tensions around (1) embedded values in LMMs, (2) AI’s influence on decision-making on farms, (3) the integration of AI in current knowledge systems by human-AI collaboration, and (4) the economics of AI-based welfare assessment and improvement. We conclude that LMMs could help automate welfare assessment and communicate results to humans in accessible formats, but outcomes depend on which stakeholders are involved in the development process. We advocate for developing AI-based welfare assessment tools through the One Welfare framework, recognizing that AI deployment affects humans, animals, and the environment simultaneously, and suggest potential pathways for animal welfare scientists to engage in the process.

## Introduction

Growing societal concerns about animal welfare (1), coupled with increased farm automation, have accelerated the development of automated welfare assessment tools (2). Previous attempts mostly used precision livestock farming (PLF) to continuously monitor and optimize environmental conditions, production, and health (3). However, PLF development often lacks rigorous validation by independent third parties, particularly across diverse contexts and environmental conditions (4, 5). While previous work emphasizes the potential benefits PLF could bring to animal welfare in theory (3), applications often fail to address the inherent complexity of welfare as a multidimensional concept that probably could not be adequately captured by a single technological solution (6). The threats of PLF for animal welfare are rarely discussed, such as inaccurate predictions stemming from poor reliability (4), reduced human-animal interactions (7, 8), or increased intensification focusing on productivity without considering welfare needs (9).

Artificial intelligence (AI), particularly computer vision, has gained significant momentum in livestock farming applications (10), including detecting diseases or monitoring animal activity patterns. Recently, the emergence of general-purpose generative AI tools, including large multimodal models (LMMs; systems trained on diverse data types such as text, images, and audio that can process and generate content across different modalities) has initiated a widespread “AI revolution” (11). A variety of foundation models pre-trained on vast amounts of unlabeled data are readily available (12). Foundation models can be adapted to diverse applications (12, 13) through fine-tuning (retraining the model on domain-specific data to optimize performance) and prompt engineering (crafting specific input instructions to guide model outputs without retraining the model itself). The development of foundation models and corresponding applications is advancing globally (e.g., USA: ChatGPT-OpenAI, Claude-Anthropic, Gemini-Google, Llama-Meta, EU: Mistral, China: DeepSeek) with the goal of creating AI agents (14) that perform tasks autonomously following human instructions. However, it is unclear if and how generative AI tools could help animal welfare.

Animal welfare science is inherently value-based (15), because farmers, scientists, and the public may hold diverse perspectives on what constitutes a good life for animals. This diversity creates challenges in establishing unanimous standards for animal welfare assessment, a problem not sparing AI-based tools. Different foundation models already vary in how they weigh animal interests (16) and evaluate animal harms (17), with some erasing the reality of intensive livestock farming (18). Developing valid, ethical, and effective AI-based welfare monitoring requires interdisciplinary collaboration (19, 20) and engagement with farmers and society (21). Scientists may underestimate uncertainties outside their expertise (22) and experience false confidence when using AI (23). Thus, without proactive involvement of welfare scientists in AI-based animal monitoring, unexamined assumptions may limit potential benefits or even cause harm to animals (24).

This paper aims to highlight potential roles for animal welfare scientists in the development of AI-based welfare assessment tools and outline how AI could be used for furthering One Welfare (improving the welfare of animals, humans, and the environment), focusing on farm animals. First, we discuss uncertainties around automated welfare monitoring. Second, we present a case study using LMMs to assess welfare indicators. Finally, we consider how generative AI could support welfare monitoring and One Welfare in practice.

## Uncertainties in automated welfare assessment

**Figure 1:**
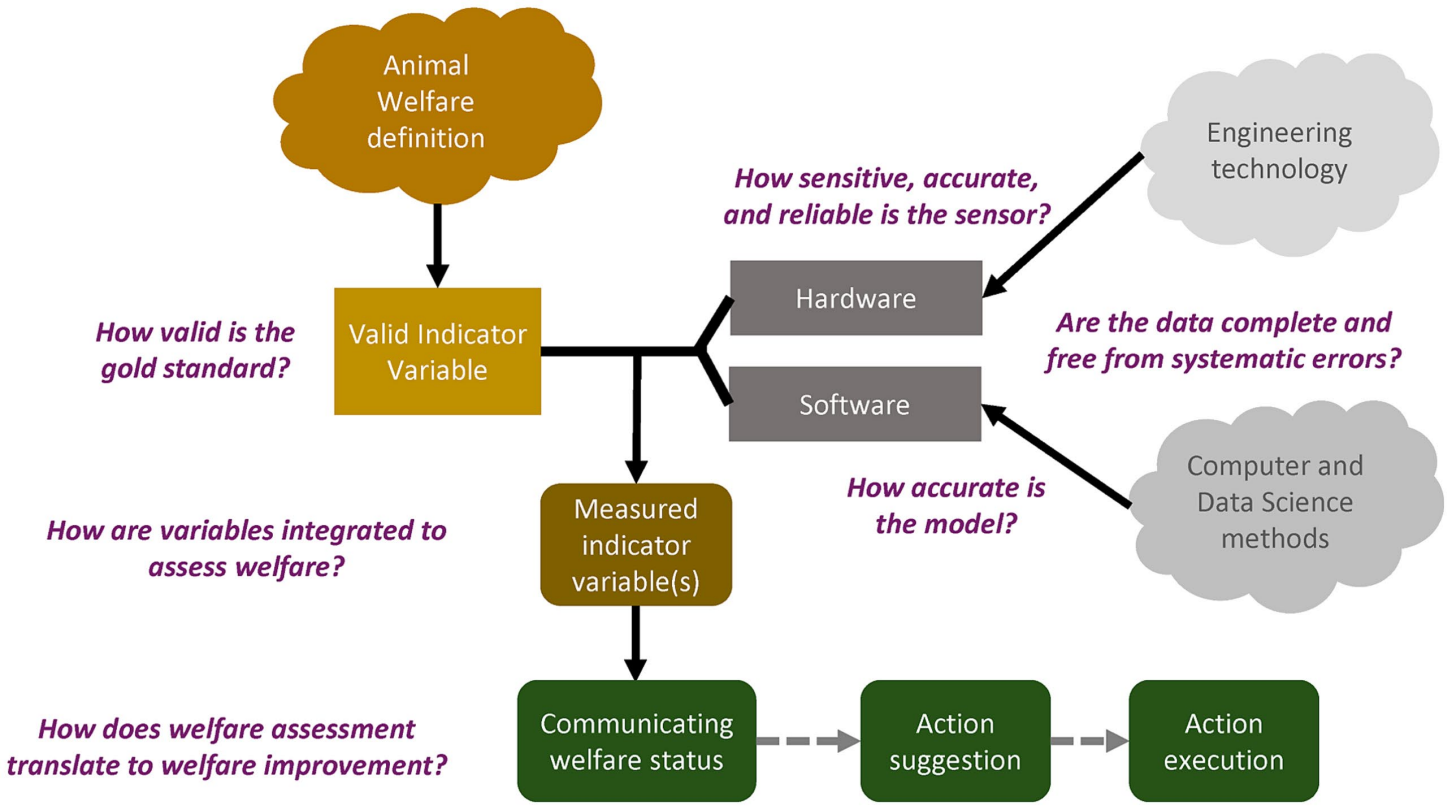
Areas (indicated by different colors) involved in the development of automated animal welfare assessment tools. Questions indicate uncertainties associated with the different components of the development process.

Automated welfare monitoring is a multidisciplinary process, requiring expertise in animal welfare, engineering, and data science with its practical impact often depending on ethical, psychological, social, and economical factors (Figure 1). The uncertainties in this process are rarely considered systematically during the development or validation of AI-based monitoring tools. How animal welfare should be assessed depends on how it is defined (e.g., (25)). Frameworks behind current welfare assessment schemes such as the five freedoms (26), three spheres (15), five domains (27), and four principles (28) combine different aspects of animals’ life (e.g., nutrition, comfort, behavior, health, naturalness, affective states) to determine welfare. Moreover, animal welfare science has evolved beyond farm-level evaluations to individual-level (29), and emphasizes evaluating affective states (30), and need for positive experiences (31).

### Uncertainty of indicators

The validity of welfare indicators may vary depending on the spatial and temporal level of analysis. For example, environmental and animal-based indicators used in the Welfare Quality protocol (WQP; e.g. cleanliness, illness signs, resource provision) can be measured in a standardized manner and have been validated to reflect farm-level welfare. However, these indicators and integration methods would need to be adapted for AI-based individual welfare assessment. It is also unclear how continuous measurements could be aggregated over time to determine animals’ quality of life (32). Current welfare assessment protocols lack validated affective state indicators. Although some sensor technology approaches have been proposed (33), these require validation (34, 35). Research still needs to identify reliable species-specific affective state indicators for on-farm use.

### Uncertainty of gold standard

Gold standards (annotated datasets with correct labels) are the foundation for training AI-based systems. Some indicator variables may be compromised by poor quality gold standards due to low intra- and inter-observer reliability (36) (e.g., lameness, ear position). Lack of diversity in gold standard dataset limits the model’s generalizability across farms, especially in behavior assessment (5, 20). Establishing robust gold standards for validated indicators may require collaboration with animal welfare scientists in curating diverse datasets.

### Uncertainty of hardware

Data quality varies when collecting data on different farms. Automated welfare assessment relies on sensor, video, and audio data, requiring high sensitivity to detect subtle changes in animals within complex environments. Data “noise” from unidentified factors (e.g., management changes) may overshadow welfare-relevant changes (37). Hardware must withstand harsh conditions (e.g., dirt, collisions) and reliably transmit high-resolution data or run local processing algorithms. These factors limit real-world applicability of experimental solutions (3). Standards for sensor accuracy, calibrations and data cleaning are required but lacking.

### Uncertainty of software

Most current AI-based welfare monitoring tools rely on traditional task-specific machine learning approaches, requiring large amounts of training and testing data (13). In agriculture, LMM use will likely increase (38) as LMMs can perform new tasks using prompt engineering without retraining (39). However, high-quality (albeit small) datasets of welfare indicator variables remain essential. Another challenge is balancing cloud-based LMM processing (requiring large data transfers) with edge-deployed smaller models that process data locally (12). However, model distillation techniques (transferring knowledge from larger to compact models) offer promising solutions (40).

### Uncertainty of integration

Although broad consensus exists on which indicators are essential for comprehensive welfare assessment, how to weigh them appropriately (i.e., valence, severity, duration of conditions) remains unresolved (41–43). Furthermore, each indicator carries measurement errors and uncertainties that accumulate when combined. Although attempts exist to establish cumulative welfare assessment frameworks (e.g., Welfare Footprint Project; (44)), differing ethical views among stakeholders may lead to varying opinions about the most important welfare indicators (15). Determining the cumulative impact of single indicators over time may be easier than aggregating different indicators into one measure.

### Uncertainty of application

The availability of reliable AI-based welfare monitoring does not guarantee system use. Without clear benefits for farmers, uptake may be limited without regulatory requirement or other incentives. Even if these tools are used, improved animal welfare is not guaranteed due to potential “rebound effects” when owners rely excessively on AI-based monitoring and reduce personal observation (45). Human action is still required for corrective actions, and whether this happens may be influenced by how the welfare status is understood and the types of actions required (46) Establishing thresholds that trigger an alarm for action may prove challenging (47), and systems may overlook concerns to reduce alarms to manageable levels. Human factors are key in improving animal welfare (48), emphasizing the need for user-centered design accounting for farmers’ motivations, values, skills, and knowledge.

## AI-based welfare monitoring – a prompt engineering case study

**Figure 2:**
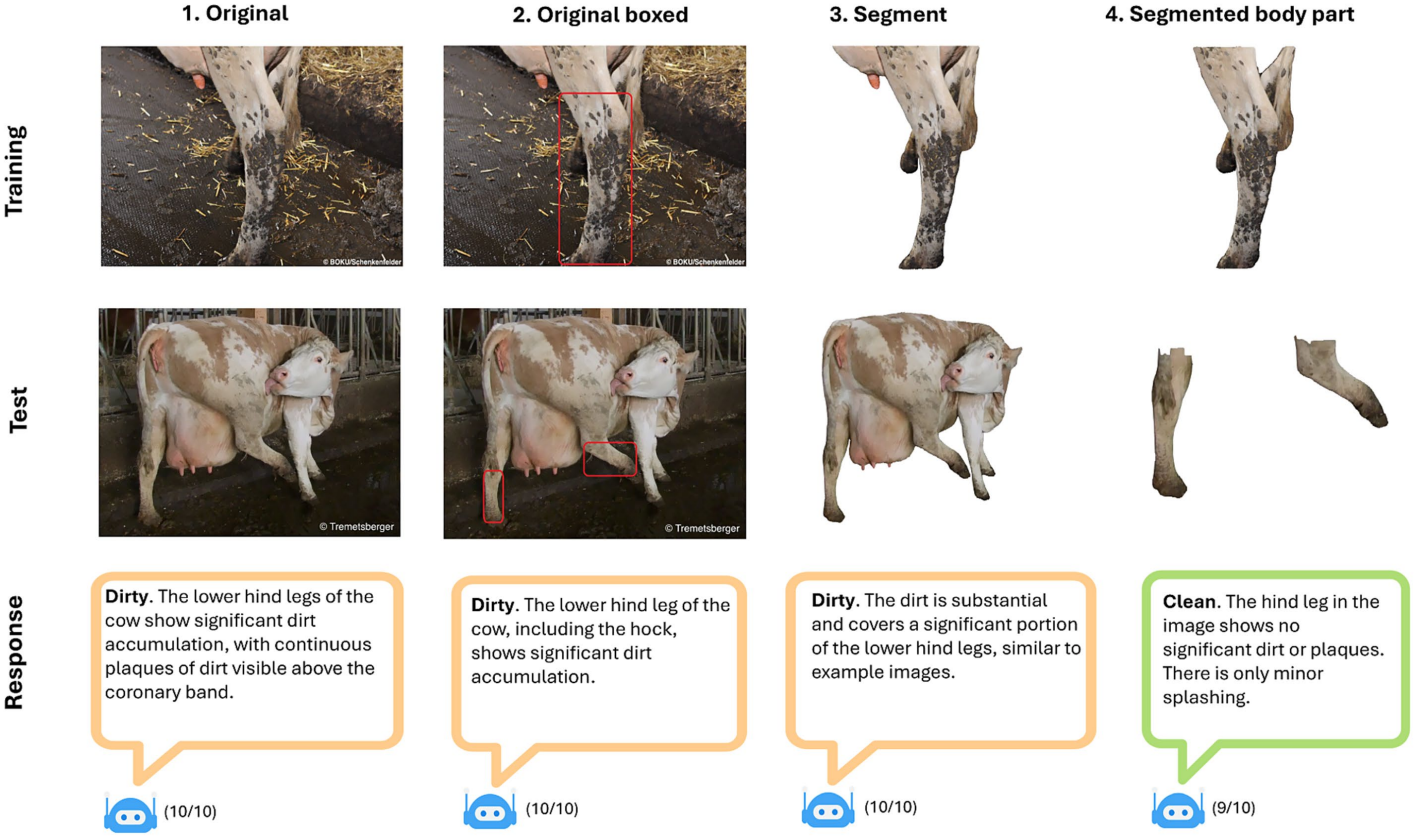
Using GPT-4o to assess cleanliness according to the Welfare Quality protocol for cattle. Columns show image pre-processing approaches. Rows show an example of training and testing images and generated reasoning responses in bubbles (orange false, green correct). The proportion of the given response out of 10 runs is shown at the bottom.

To demonstrate the potential application of LMMs for assessing welfare indicators through prompt engineering, we present a case study. We employed GPT-4o (OpenAI) through Application Programming Interface calls to evaluate cattle cleanliness by categorizing three body parts (hind leg, hindquarter, udder) as either clean or dirty, following the WQP. We used a system prompt instructing GPT-4o to act as an experienced animal welfare scientist with 20 years of farm audit experience. We then provided WQP instructions and implemented few-shot prompting by showing the model two dirty and two clean example images for each body part. Our test dataset comprised eight images per body part (four dirty, four clean) from auditor training materials, with labels by 2 experts (Tierwohltraining, BOKU University, Vienna). We also compared four pre-processing methods: (1) original image (control), (2) bounding box around the body part of interest, (3) segmented image with background removed, (4) segmented image showing only the body part of interest. To account for the probabilistic nature of generative AI, each image was assessed 10 times, with the model explaining its reasoning (Figure 2). Results showed that using segmented images of specific body parts yielded the best performance, with moderate accuracy (hind leg: 0.71, hindquarter: 0.62, udder: 0.52). However, precision was lower (hind leg: 0.63, hindquarter: 0.57, udder: 0.52) and recall of “dirty” images was almost perfect (hind leg: 1, hindquarter: 1, udder: 0.75).

For the image material used, we achieved a Kappa of 0.5 for segmented hind legs, representing an acceptable level of agreement for human observers. However, the model was biased toward labeling images as “dirty” and removing the background (often containing dirt) brought little improvement. This likely stems from LMMs’ difficulties with spatial reasoning (49) and subsequent challenges in identifying specific body parts for assessment. Focusing the model’s attention using segmentation improved performance. In addition, brown coat pattern and overlapping body parts (e.g., tail over hind leg in Figure 2) may also have biased the model toward classifying images as dirty. We examined only one welfare indicator using a limited image set with varying quality and non-standardized viewing angles; addressing these limitations could potentially enhance performance. However, LMMs sometimes struggle with tasks that humans find trivial (e.g., reading clocks (50)). How well this approach generalizes to other WQP indicators could be further investigated along with a comparison between different LMMs.

LMMs could help scaling up existing protocols, creating new ones (e.g., assessing affective states by decoding vocal and visual signals), and communicating results to humans in a relatable way. However, as our results with cleanliness as one of the “simplest” indicators in WQP demonstrate, current LMMs still require fine-tuning for acceptable accuracy. This could be achieved by providing domain-specific data to open-source LMMs or by utilizing Retrieval Augmented Generation (RAG, see (13) for an example) to provide context-specific materials during prompting.

## Discussion

### AI for welfare assessment

Due to the outlined uncertainties during development, outcomes from AI-based welfare assessment depend critically on which stakeholders drive the process. On one hand, if development is driven mainly by entities with purely commercial interests, it risks (1) scaling unvalidated metrics that lack animal welfare relevance, (2) further intensification for profit with lower or stagnating welfare standards, (3) not using AI’s potential to improve animal welfare beyond productivity improvements, and (4) restricting farmer and public authority access to data due to proprietary algorithms. On the other hand, if development is driven mainly by entities with unilateral interests in animal protection, it risks (1) scaling up novel assessment methods (e.g., affective states) without sufficient validation, (2) creating solutions disconnected from practical farming realities, leading to frustration and disinterest among farmers, (3) resulting in high-price niche-products, and (4) limiting innovation from commercial companies. The involvement of animal welfare scientists in the tool development and AI regulation process could mitigate some of these risks (e.g., validity concerns) but pathways for engagement are often lacking.

### AI for One Welfare

It has long been recognized that the welfare of animals is interconnected with the wellbeing of humans and the environment (24, 51). We argue that AI-based welfare assessment tools should be developed with a One Welfare mindset, a holistic approach reflecting that the spread of AI simultaneously impacts human and animal lives and the environment.

#### Who tells AI what is right and wrong?

As AI-tools become prevalent, the messages applications convey to users (or the actions AI agents may take) can have a profound impact. ChatGPT can influence users’ moral judgments, despite their awareness of interacting with a chatbot (52), indicating the potential for biased decisions in the context of livestock farming when users engage with AI systems. This highlights not only the need for user education, but also the importance of AI alignment (ensuring that AI reflects and upholds essential values). However, values in society around the use of animals are not homogenous and animal interests are currently not explicitly considered in AI alignment (53). Current LMMs are not autonomous moral agents but their training data and guidelines influence moral judgments. AI-based welfare assessment tools may implicitly or explicitly follow certain ethical guiding principles (e.g., anthropocentric, pathocentric, biocentric, utilitarian (54, 55)). Livestock farming is tied to complex ethical considerations which may vary based on regions and farming systems. Thus, AI-based welfare assessment needs to be transparent about ethical principles in the model and may require a “practical” ethical approach (56), which is bottom-up (i.e., based on real-life observations) rather than top-down (i.e., based on specific theories). Such an approach may help conscientious people, organizations, and policy makers address nuanced, real-life problems that balance animal welfare, rural poverty, environmental sustainability, and food availability (56–58).

#### Who decides what to do?

Technology use and human-animal contact vary across farming systems but currently practical improvements to farm animal lives depend on human decisions. However, some barns may be managed without human presence in the future, a kind of “better wilderness” (59) where animals are cared for and looked after by AI. While humans hold responsibility in current farming systems, they are increasingly supported by data. Nevertheless, rapid digital technology adoption, e.g., in the dairy industry (60), has left many farmers struggling to interpret complex data. LMMs could address this challenge by providing simple explanations of technical information and personalized suggestions for improvement. Decisions impacting animal welfare happen at multiple levels and “decision makers” are a diverse group including farm workers, farm owners, veterinarians, company representatives, and private consumers whose decisions may include making tradeoffs between their own interests, animal welfare, and environmental impact (61). Complex psycho-social processes are also involved in decision-making, which may modulate the interaction between AI tools and the human end user, ultimately leading to variable welfare outcomes (62). Sustainable solutions will require ethical AI frameworks and regulations, clarity about accountability (63), data privacy, and a tailored communication approach recognizing diverse cultural and personal values while mitigating bias without propagating unrealistic outcomes (18).

#### Where does AI fit in current systems?

Decisions on farms are results of knowledge systems based on farmer experience and formal training, and that often include advisors (e.g., veterinarian, nutritionist, accountant; (64)). Completely outsourcing welfare assessment to AI is unlikely to be beneficial as diversity in “ways of knowing” strengthens decision-making processes (65). However, integrating LMMs to decision-making systems as an “artificial way of knowing” (i.e., a perspective based on high-resolution longitudinal datasets beyond the scale of human comprehension), and using AI as a “copilot” for farmers could bring enormous benefits (see (66) for a human medicine example). Examples for using AI as agricultural advisor already exist (67) and these approaches could be further developed and integrated with “human-in-the-loop” solutions (68) and farm-specific data to provide region- and context-specific advice in multiple languages, independent of literacy.

#### Who will pay for it?

Willingness to financially support a good life (69) for farm animals varies greatly among individuals and societies. Replacing livestock farming with alternative proteins is still in its infancy (70) and may not always be culturally accepted or possible. Establishing high-welfare farming systems while ensuring food security across different cultures and income levels is challenging as the willingness or capability of farmers to improve animal welfare may be limited. Improving welfare standards only with price increases had limited success due to the “consumer-citizen gap” (discrepancies between consumer behavior and attitudes, (71)) and the lack of a sizable uptake that would financially support transformative change (72). Realizing animal welfare standards that are aligned with societal values could be financed centrally (73), similarly to other subsidies to animal agriculture. However, this would require strong political will and meaningful ways to translate animal welfare measures to policy, an area requiring further involvement from animal welfare scientists (74). AI-based welfare assessment may also involve social (e.g., job displacement) and environmental costs (e.g., energy usage, rare-earth mining for computer chips) that should be considered through a One Welfare approach. For both data privacy and reduced energy consumption, deploying small-sized LMMs locally on edge devices would be ideal moving forward.

#### What could animal welfare scientists do?

Many tools are already marketed for automated welfare assessment without external validation (4, 5) and development in AI applications is quicker than what many animal welfare scientists and legislators are used to. Here we suggest some actions for animal welfare scientists to contribute to the development of reliable AI-based welfare assessment tools: (1) Delivering accessible communication materials for non-experts to understand the strengths and limitations of welfare indicator variables (e.g., level of evidence and confidence for a variable in a species), (2) Establishing rigorous quality standards for sensor data and indicator variables (75), (3) Supporting the creation of certification schemes for commercial AI-based welfare assessment tools, (4) Advocating for the systematic consideration of uncertainties during the welfare assessment tool development and validation process, (5) Publishing high-quality, annotated, and open-sourced indicator variable datasets along with detailed descriptions (e.g., ethograms, relationship between indicator and welfare) to be used as gold standards and for synthetic data generation (76), (6) Consulting and publishing position papers on incorporating animal welfare into AI development and regulation (e.g., EU AI Code of Practice (77)).

## Conclusion

AI-based welfare assessment tools may bring individualized welfare assessment, which could help providing a good life for farm animals under human care. However, critical uncertainties persist regarding the validity of measurements, methodological approaches, and how end-users interpret and implement the resulting information to improve animal lives. Thus, it is time for animal welfare scientists to get involved in the development and validation of AI-based tools for welfare assessment and improvement in the spirit of One Welfare. This will ensure systems that respect animals along with the wellbeing of farmers, consideration of consumers, and environmental impact.

## Data Availability

Publicly available datasets were analyzed in this study. This data can be found here: https://tierwohltraining.boku.ac.at/home/uebungszentrm/quiz-uebersicht-rinder/. Code for the case study can be found under the following link: https://github.com/skysheng7/welfare_assess_GPT4o.git.
